# Inclusion of climate change and planetary health in masters of public health curricula in the UK

**DOI:** 10.1093/eurpub/ckaf158

**Published:** 2025-09-13

**Authors:** Ana-Catarina Pinho-Gomes, Cecilia Sorensen, Danielly de Paiva Magalhães, Shakoor Hajat, Harry Rutter

**Affiliations:** The George Institute for Global Health, Imperial College London, London, United Kingdom; Institute for Global Health, University College London, London, United Kingdom; Global Consortium on Climate and Health Education, Global Consortium on Climate and Health Education, Mailman School of Public Health, Columbia University, New York, NY, United States; Emergency Medicine, Columbia Irving Medical Center, Columbia University, New York, NY, United States; Global Consortium on Climate and Health Education, Global Consortium on Climate and Health Education, Mailman School of Public Health, Columbia University, New York, NY, United States; Emergency Medicine, Columbia Irving Medical Center, Columbia University, New York, NY, United States; Centre on Climate Change and Planetary Health, London School of Hygiene and Tropical Medicine, London, United Kingdom; Department of Social and Policy Sciences, University of Bath, Bath, United Kingdom

## Abstract

Due to the many health impacts of climate change, it is imperative to equip public health professionals with the skills and knowledge to work on climate mitigation and adaptation. However, it is unclear to what extent Masters of Public Health (MPH) include climate change and related subjects in their curricula. A survey was sent to MPH directors in the UK with questions about inclusion of climate change and related subjects in the curriculum. Russell group universities and those commissioned by NHS England Workforce, Training and Education were invited to take part. A total of 27 MPH courses were included (100% response rate). Climate change and related subjects were included in optional or core modules on other subjects, with health protection and health improvement being the most common. Two MPHs had only one lecture/seminar on climate change and one MPH did not cover these topics in the syllabus. The most common subject included in curricula was climate change (24, 89%). Most MPH directors wanted to increase the inclusion of climate change and planetary health in the curriculum (12, 55%) but could not do so due to lack of space within an already overloaded curriculum (10, 37%). Despite the recognition of the importance of climate change and health education by MPH course directors, the inclusion of those subjects in curricula remains variable and not as thorough as required given the importance of the topic. Addressing barriers is warranted to enable public health professionals to gain the required skills in climate mitigation and adaptation.

## Introduction

Climate change has been described by the Director General of WHO as “one of the greatest health threats for humanity” [1], with worsening impacts affecting generations to come [2]. Climate change has multiple and complex effects on population health. Direct consequences include mortality and morbidity due to extreme weather events, such as heatwaves, floods, droughts, and storms [3–5]. Indirect consequences include food insecurity, violence and armed conflicts, migration, loss of livelihoods, vector-borne diseases, and destruction of ecosystems [6–9].

As with other public health crises, the consequences of climate change are disproportionately experienced by the most disadvantaged and vulnerable populations both within and between countries [10, 11]. Climate change is already exacerbating existing health inequalities, and will continue to do so unless appropriate adaptation measures are adopted in a timely and equitable manner. Furthermore, climate change is a system stressor that compounds other societal challenges, such as poverty, and strains economic and societal welfare [12].

Our understanding of the impact of climate change on health systems remains limited. There is some evidence demonstrating that extreme weather events cause surges in healthcare demand, which are difficult, if not impossible, to meet due to lack of human and financial resources as well as compromise of healthcare facilities [13–15]. Developing health systems that are resilient to the myriad impacts of climate change is critical to avoid both increases in morbidity and mortality and health system collapse. Furthermore, health systems account for about 5% of global greenhouse gas emissions and contribute to environmental degradation throughout the lifecycles of pharmaceuticals and medical equipment [16].

In this scenario, training public health professionals in the nexus of climate change and population health is crucial. Their knowledge and experience of dealing with complex systems and addressing entrenched health inequalities is instrumental to building climate-resilient health systems and developing policies that support populations in proportion to their needs and resources. For instance, health professionals have a key role to play in lowering the greenhouse gas emissions of health systems by investing in primary prevention and hence reducing the need for healthcare, developing effective surveillance mechanisms and early warning systems to protect population health from extreme weather events, and leading the emergency response to climate-related hazards, in collaboration with community organizations, to support affected populations [17].

The recognition of the myriad consequences of climate change on health led to the creation of the Global Consortium for Climate and Health Education (GCCHE) in 2017. The vision of the GCCHE, which has 400 members across 75 countries, is that all health professionals throughout the world—doctors, nurses, public health practitioners, mental health practitioners, and allied health specialists—will work together to prevent, reduce, and respond to the health impacts of climate change. To achieve this ambitious vision, the GCCHE seeks buy-in from deans or heads of school and has been organizing free online courses to educate health professionals in partnership with other organizations. For instance, it co-led the European Responder Course on Climate and Health with the Association of Schools of Public Health in the European Region (ASPHER) in 2024. There have been other courses in Africa, South America, the Caribbean, and Southeast Asia. The courses are important to health students and professionals worldwide but especially in low and middle-income countries, which include some of the countries experiencing the most severe impacts of climate change on population health and lack the resources to train sufficient health professionals and develop resilient health systems [18].

The GCCHE courses are mapped to the core competencies to prepare health professionals to respond to the climate and health crisis [19]. These are organized into five domains: knowledge and analytical skills, collaboration and communication, policy, public health practice and clinical practice, and include both key concepts and learning objectives. In recognition of the importance of climate change for public health, the Association of Schools of Public Health in the European Region has published Climate and Health Competencies for Public Health Professionals [20]. These are aligned with the GCCHE competencies and are similarly organized in domains: knowledge and analytical skills, communication and advocacy, collaboration and partnerships, and policy. There is, though, a greater focus on the European context, vulnerability, and health and political systems. The updated Public Health curriculum, which informs MPH curricula across Europe, also includes competencies related to climate and environmental change in recognition of the interdependencies and interactions between population and planetary health, i.e. the wider ecosystems on which population health depends [21].

Climate change is a relatively recently recognized public health challenge and hence it did not feature in the training of previous generations of public health professionals who are now training current and future generations. Therefore, faculty may lack the knowledge and skills to train students [22]. It has not been routinely included in the curricula of Masters of Public Health (MPH) programs, and does not constitute a major portion of the training curriculum of the Faculty of Public Health (FPH), the professional body that is responsible for the training of public health specialist doctors and professionals in the UK.

However, the increasing recognition of climate change and planetary health (i.e. the health of human civilization and the natural systems on which it depends) as major public health challenges has led to the inclusion, albeit to a variable extent, of these topics into the curricula of many MPH programs [23]. As completion of this degree is a requisite of Public Health training according to the FPH curriculum, it is important to understand to what extent knowledge and skills in climate change and health are being developed during MPH courses to identify gaps that need to be addressed by other training activities. Surveys to faculty have previously been used to map curricula and audit the content of MPH [24, 25]. This study developed and administered a survey to [1] document how climate change and planetary health were covered by MPH and equivalent degrees offered by universities across the UK, and [2] make recommendations to MPH courses on incorporating climate change and health content into their curricula.

## Methods

### Study design

A survey was sent by email to the course directors of MPH and equivalent degrees offered by a sample of UK universities in October 2024 (details on eligibility criteria provided in the population section). The survey included questions about how each program covered climate change and associated subjects, such as planetary health and one health (full questionnaire available in Supplementary Data). There were also questions about barriers and facilitators to increase the coverage of these subjects. The survey questions are provided in Supplementary Data. MPH course directors were contacted by email and invited to take part. Follow-up emails were sent until responses from all courses were obtained. There was a clear notice at the beginning of the questionnaire stating that participation was taken as consent for data to be shared in scientific publications or presentations.

### Population

The sample included the Russell group universities as well as the universities commissioned by NHS England Workforce, Training and Education as part of the specialty training in Public Health Medicine. MPH course directors were contacted by email and invited to take part by answering the survey. Universities that are part of the Russell Group but do not offer an MPH or equivalent were excluded. The Russell Group represents 24 research-focused universities located in every region and nation of the UK. They produce more than two-thirds of the world-leading research produced in UK universities. The list of included MPH programs is provided in Supplementary Data. Further information about the Russell Group is available on their website [26].

### Data analysis

Frequency tables were used to summarize answers to multiple-choice questions about the current and desired inclusion of several climate change related subjects. Content analysis, specifically conceptual analysis, was undertaken by one researcher (ACPG) to summarize answers to open-ended questions about barriers to inclusion of climate change and related subjects in MPH curricula [27]. The conceptual analysis involved investigating the occurrence of explicit themes and modules related to climate change and planetary health in MPH curricula.

## Results

**Figure 1.**
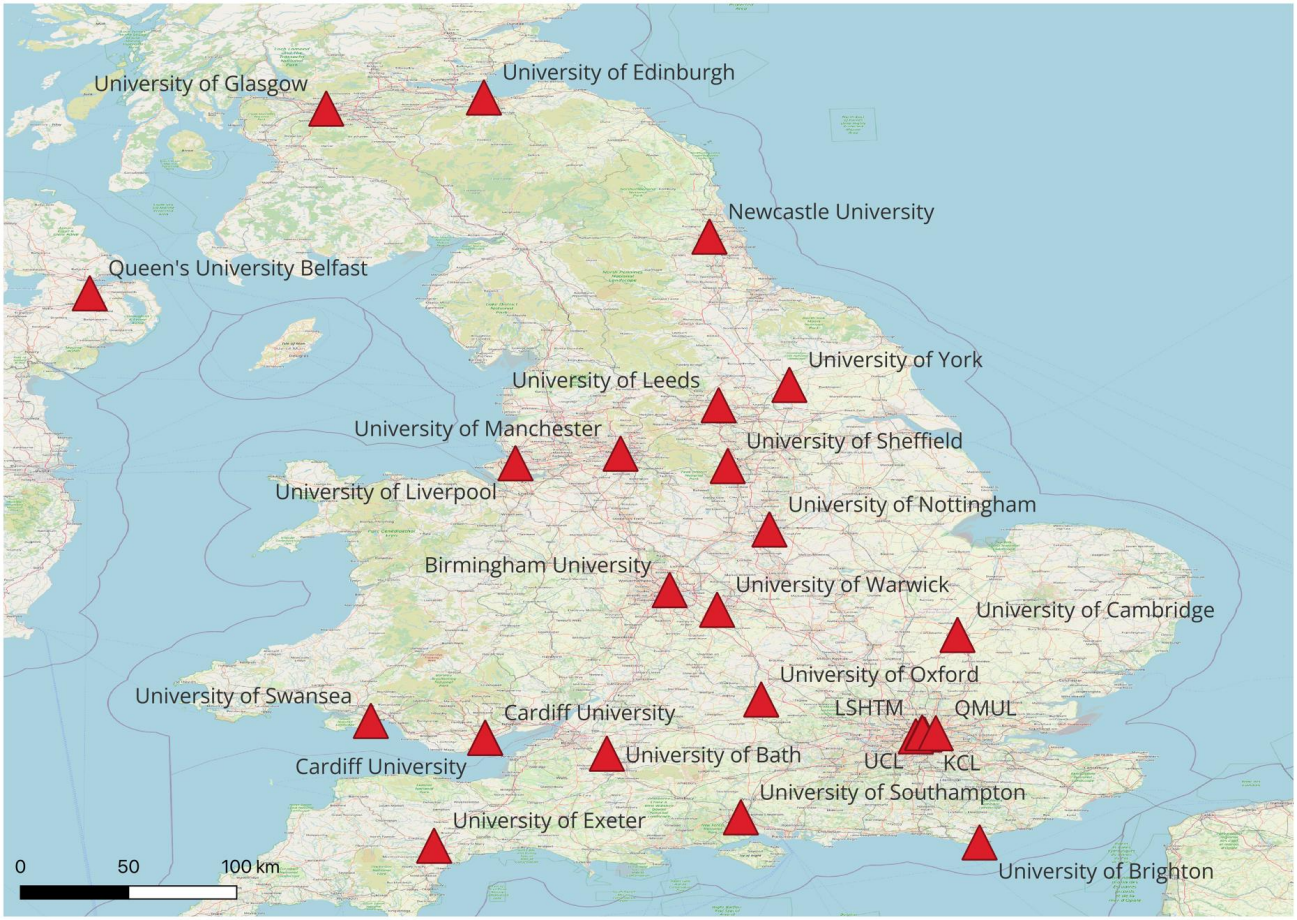
Map of the universities included in the survey.

All 27 MPH courses provided by Russell group universities or commissioned by Health Education England answered the survey, a response rate of 100%. The geographic location of the included universities is displayed in Figure 1. These included 23 on-campus programs, three online programs, and one hybrid program. Two universities which offered on-campus programs additionally had an online MPH with a slightly different curriculum.

### Quantitative analysis

Inclusion of climate change in one or more modules on other subjects (e.g. health protection, urban health) was the most common (*n* = 16, 59%). An optional module focused on climate and health or related subject was offered by five MPH programs, whilst three programs had a core module on climate change. Two degrees only had one lecture/seminar on climate change and one degree did not cover this topic in the syllabus. No MPH programs offered a bespoke specialization on climate change.

When asked whether climate change and planetary health should be part of MPH curricula, there were mixed views. Twelve course directors (44%) considered that these topics should be covered in core modules and eight as optional modules. Three (11%) were uncertain and the remaining four considered that climate change should be woven throughout the curriculum as it “transcends most of public health” and “is too important to be left to a single module or to optional modules that students may miss.”

**Figure 2.**
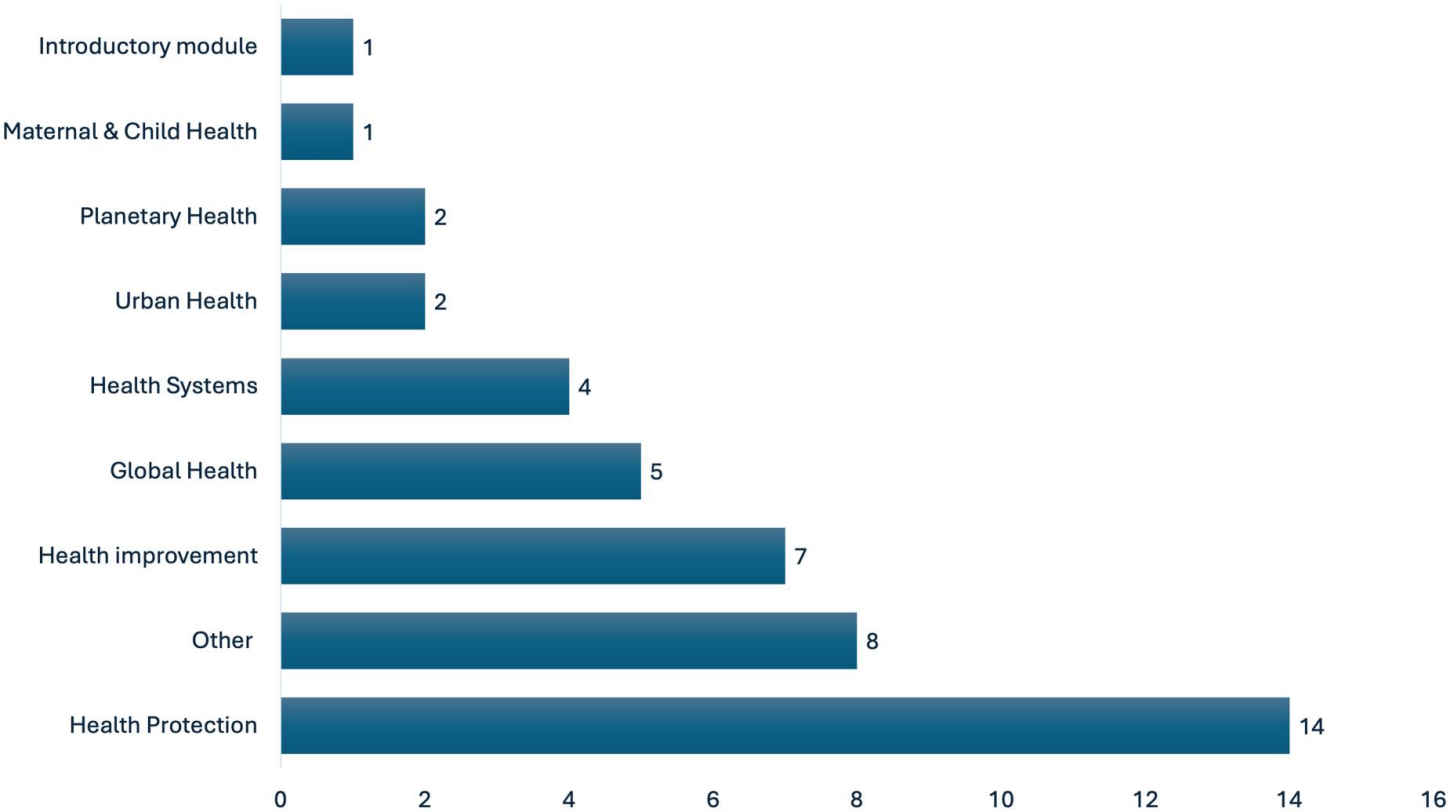
Modules in which climate change and related subjects are covered in the curriculum. The numbers represent the number of MPH programs that covered that subject (*N* = 27).

**Figure 3.**
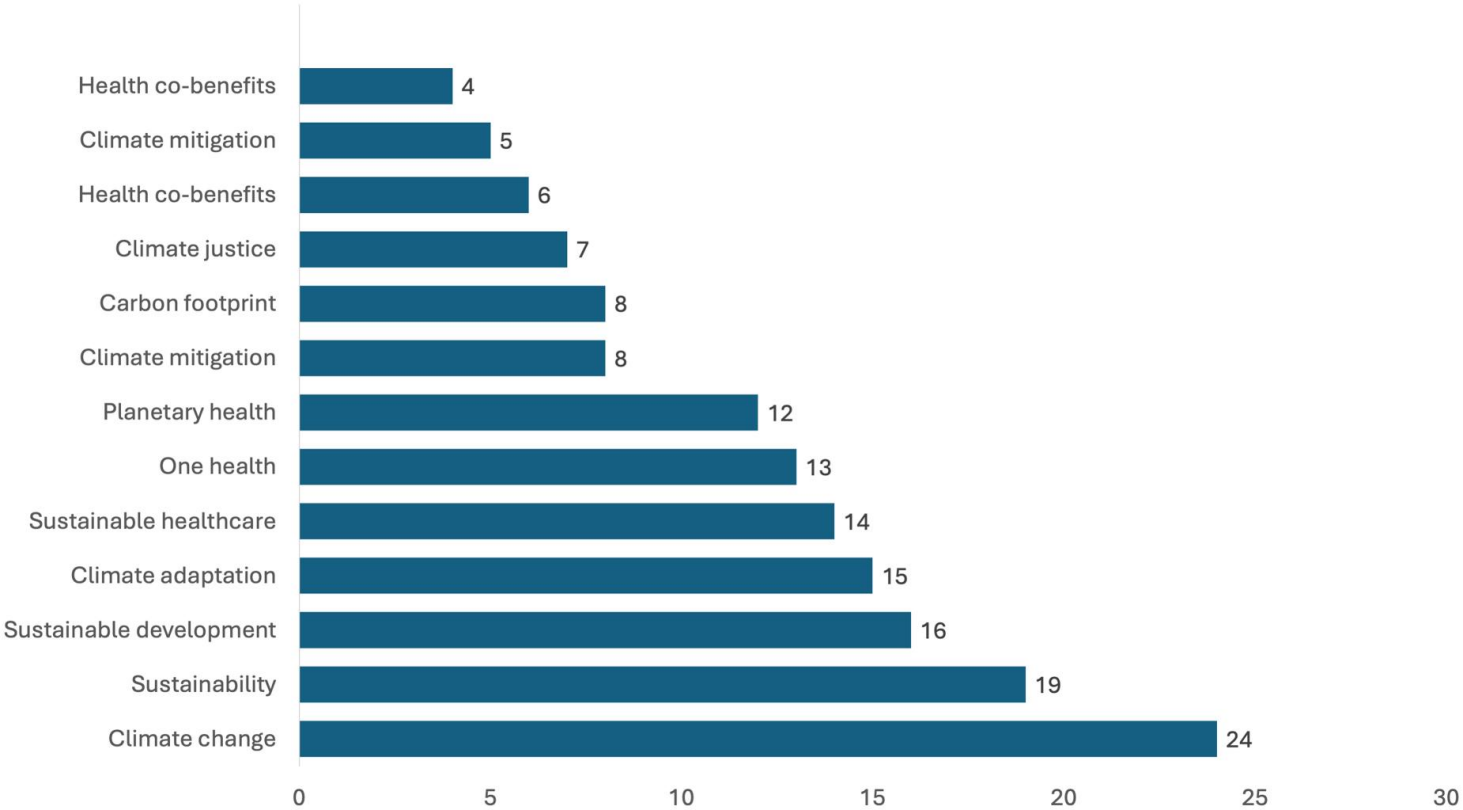
Coverage of climate change and related subjects by MPH curricula. The numbers represent the number of MPH programs that covered that subject (*N* = 27).

Climate change and related subjects were covered in a variety of modules. The most common module in which climate change was included was health protection (14, 52% of the MPH programs) followed by health improvement (7, 26%), global health (5, 19%), and health systems (4, 15%) (Figure 2). Climate change was also covered by urban health (2, 7%), planetary health (2, 7%), and maternal and child health (1, 4%). The most common relevant subjects included in the curriculum were: climate change (24, 89%); followed by sustainability (19, 70%); sustainable development (16, 59%); climate adaptation (15, 56%); and sustainable healthcare (14, 52%). The remaining subjects were covered by less than half of the programs (Figure 3).

Most course directors stated that they would definitely like to increase the inclusion of climate change and planetary health in the curriculum (12 of 27, 55%), with seven (19%) uncertain about it. The main barriers to increase the coverage of climate change and related subjects in the curriculum were the lack of space in an already full curriculum due to the breadth of subjects covered by Public Health (10 of 27, 37%), followed by lack of staff or staff time to develop new content (7 of 27, 26%) and lack of expertise (4 of 27, 15%).

### Content analysis

One respondent mentioned that environmental health is considered low priority in comparison to individual health. To address barriers to climate and health curricular integration, two respondents mentioned that they are trying to integrate climate change into other modules whenever relevant, collaborating with other departments or inviting guest lecturers to complement the expertise of the MPH teaching team in climate change as this is “not a traditional public health subject”. Offering optional modules was another strategy proposed to add new content by eight course directors, but with a syllabus broader than climate change. However, another course director mentioned that the number of students limited the possibility of offering new optional modules.

## Discussion

This study found that climate change and related subjects are covered to some extent by almost all MPH curricula in the UK. The most common topic addressed is climate change itself, and the most common module within which it sits is health protection. The emphasis on health protection suggests a focus on preventing and managing the impacts of climate change, such as disease outbreaks, extreme weather events, or environmental hazards. This reflects a strong alignment of these UK public health education with global health priorities, particularly the need to prepare professionals for the challenges posed by climate change. Despite recognizing the importance of the subject, less than half of MPH course directors considered that climate change should be included in core modules. Only 55% of course directors stated an interest in increasing the inclusion of climate change and related subjects (e.g. planetary/one health, sustainable development) in the curriculum. Several challenges were identified as being behind this, including overcrowded curricula, and a lack of expertise on a topic that is not yet considered a part of core public health education. Strategies to add climate change to MPH programs included offering optional modules on climate change and inviting external speakers with expertise on the subject.

The finding that all bar one MPH programs covered climate change and/or related subjects is higher than the 70% reported for Masters level programs in Europe by an international survey [28]. This international survey received responses from 279 (22%) of 1251 institutions in 81 (59%) of 138 countries. 196 (70%) of 279 responding institutions and 62 (77%) of 81 responding countries reported providing climate and health education during 2023–24. The number of responding institutions providing climate and health education was 53 (80%) of 66 in the European region, 21 (72%) of 29 in the Western Pacific region, 5 (71%) of 7 in the South-East Asia region, 97 (68%) of 143 in the region of the Americas, 15 (63%) of 24 in the African region, and 5 (50%) of 10 in the Eastern Mediterranean region. Two-hundred ninety-eight degree-level public health programs were identified during 2023–24, of which 171 (57%) reported that climate and health education was part of the required curriculum. Master’s degree programs provided the most climate and health education. A search of 135 additional non-responding institutions indicated that 36 (27%) likely offered climate and health education. A previous survey to members of the Association of Schools of Public Health in the European Region (ASPHER) had also shown that 64% of the responding schools provide climate-health educational offerings, while 63% consider these for the future [29]. However, most climate actions taken by the schools were *ad hoc* actions and a systematic approach was lacking. Overall, our findings are aligned with previous surveys and demonstrate increasing awareness of the population health impact of climate change and the health co-benefits of climate mitigation (e.g. reduction in air pollution, increasing physical activity). However, climate and health education is currently unable to meet the actual needs of public health professionals, despite the growing demand from public health graduates [29].

There was variety in how climate and planetary health were covered by MPH curricula, which may be related to the lack of familiarity and, hence, confidence in teaching these subjects. This was acknowledged by public health professionals, and specifically academic public health professionals, which is in keeping with previous evidence [30]. There is, though, increasing recognition of its importance *vis-à-vis* its impact on many dimensions of public health and potential to exacerbate inequalities within and between countries [11]. To bridge the gap between interest and skills, there is a need for targeted professional development and training for academic staff. Universities have a key role to play by offering training and encouraging knowledge exchange between institutions at national and global level [31, 32].

Although there was broad agreement about the importance of including climate change in MPH curricula, many identified curriculum overload and lack of expertise and time among teaching staff to develop new content in this area. This is in keeping with evidence from other countries and is a consequence of the gradual expansion of public health knowledge and skills [33]. Climate change, due to its impact on key public health areas, such as health protection, health improvement, and healthcare public health, should be covered by the core curriculum [34]. An alternative approach, recognized by MPH course directors, is the potential to use optional modules to add new content to the curriculum and give students the opportunity to specialize their degree in this specific area. Different approaches may be selected by different programs and this may influence students’ preference for specific MPH programs or modules.

The need to equip public health professionals with knowledge and skills to play an active role in climate adaptation and mitigation led the Association of Schools of Public Health in the European Region (ASPHER) to publish the Climate and Health Competencies for Public Health Professionals in Europe in 2021 [35]. GCCHE also published core climate and health competencies for health professionals to clarify the level and breadth of skills required of health professionals in 2023 [19]. However, most schools still do not meet those standards, with key gaps in policy, communication, and advocacy. This might be due to the slow pace of curriculum change in European universities. To develop a competent public health workforce, MPH degrees need to rapidly update their curricula and ensure they meet with European and international standards.

The multiple and varied impacts of climate change mean that students and professionals from fields other than public health also have an urgent need for climate education [36]. Climate change not only has direct health impacts (e.g. deaths cause by climate-related extreme weather events) but also indirect health impacts, mediated by the wider determinants of health, such as housing and living conditions, education, employment, built and natural environment among others. It is thus necessary to update other curricula beyond those for MPH courses to ensure that all degrees consider the direct and indirect impacts of climate change in their field and how their sector can meaningfully contribute towards climate mitigation and adaptation. Of note, some universities are now delivering dedicated Masters programs on climate change and planetary health.

Climate change education is especially important to counter dis- and misinformation that hinders the much-needed intersectoral action and political engagement to prepare contemporary societies to the multiple challenges brought in or exacerbated by climate change [37]. Only through the development of a generation of informed professionals across a variety of fields from economics to management, urban planning, engineering, architecture, agriculture, business, and science can society thoroughly assess the complex and interacting impacts of climate on diverse domains of societal wellbeing and welfare and prepare adequately to avoid or, at least, reduce their consequences [38]. Other countries in Europe should conduct similar analyses to ensure that the European Public Health workforce is well equipped to address the multiple impacts of climate change on population health *vis-à-vis* European vulnerability.

### Limitations

Although this survey investigated the extent to which climate change and related concepts are covered by MPH curricula offered by the leading Universities in the UK, we did not assess the wide range of other degrees that address aspects of climate change and sustainability. It is possible, albeit unlikely, that including those degrees would have provided a different overall picture. It is also important to acknowledge that the current study relies on self-assessment, which may not truly reflect how different MPH programs compare due to subjective understanding of concepts. It would be important to conduct a broader assessment of how climate change education is being delivered across Europe and more broadly worldwide to share good practice and ensure teaching staff become familiar with the new competencies.

## Conclusion

The need to equip MPH graduates with knowledge and skills in climate change, planetary health and related subjects is consensually recognized by MPH course directors in the UK. Despite the publication of climate and health competencies by international organizations, the inclusion of climate and health in curricula remains variable and may not be as thorough as required by the magnitude of the climate crisis. Addressing barriers is warranted to enable all students to acquire the desired level of knowledge and competency in climate mitigation and adaptation.

## Supplementary Material

ckaf158_Supplementary_Data

## Data Availability

All day are available by request from the corresponding author. Key points In the UK, climate change and health are mainly covered by MPH modules on other subjects, such as health protection and improvement. Most MPH directors agree on the need to increase inclusion of climate change and related subjects in the curriculum, but curriculum overload prevents them from doing so. Addressing barriers such as curriculum overload and lack of staff time, and expertise is imperative to enable public health professionals to acquire the skills required to play a key role in climate adaptation and mitigation.
