# Supplementary material for: Inclusion of climate change and planetary health in masters of public health curricula in the UK
Source: Eur J Public Health. 2025 Sep 13;35(6):1156–61. doi: 10.1093/eurpub/ckaf158 (PMC12707511; doi:10.1093/eurpub/ckaf158)
Supplement: ckaf158_Supplementary_Data [file ckaf158_supplementary_data.docx]

**Supplementary data**

**Questionnaire**

1.Email address. Single line text.

Enter your answer

2.University. Single choice.

- University of Swansea
- Cardiff University
- University of Cambridge
- QMUL
- University of Liverpool
- University of Warwick
- University of Bath
- UCL
- Queen's University Belfast
- KCL
- LSHTM
- Birmingham University
- Imperial College London
- University of Southampton
- University of Sheﬃeld
- University of Brighton
- Newcastle University
- University of Edinburgh
- University of Exeter
- University of Oxford
- University of York
- Durham University
- University of Glasgow
- University of Leeds
- University of Manchester
- University of Nottingham
- Other:

3.MPH type. Single choice.

- Online
- On campus
- Hybrid

4.Which of the following topics are covered in your MPH? Multiple choice.

- Planetary health
- One health
- Climate change
- Sustainability
- Sustainable development
- Sustainable healthcare
- Climate adaptation
- Climate mitigation
- Health co-benefits
- Climate justice
- Carbon footprint
- Other
- All of the above
- None of the above

5.How is climate change covered in your MPH? Single choice.

- Not covered
- Specific core module
- Specific optional module
- Included in one or more modules on other subjects
- One lecture/seminar
- Other:

6.If climate change is included in one or more modules, please indicate which modules. Multiple choice.

- Health protection
- Health improvement
- Healthcare public health
- Urban health
- Not applicable
- Other

7.Do you consider that climate change and planetary health should be part of MPH curricula? Single choice.

- Yes, as optional module
- Yes, as core module
- Maybe
- No

8.Would you like to add or increase the representation of climate change and health in your MPH? Rating.

1 Definitely not

2

3

4

5 Definitely yes

9.To what extent does your faculty feel comfortable teaching climate change and health? Rating.

1 Not at all

2

3

4

5 Very comfortable

10.What are the main barriers to including climate change and planetary health in your curriculum? Multi Line Text.

Enter your answer

11.Any further comments?

Enter your answer

**Universities included in the survey**

**Russell group universities**

- Queen’s University Belfast
- University of Leeds
- University of Manchester
- University of Sheffield
- University of Birmingham
- University of Bristol
- University of Cambridge
- Cardiff University
- University of Edinburgh
- University of Glasgow
- University of Exeter
- Imperial College London
- King’s College London (online and on campus)
- University of Liverpool
- Newcastle University
- University of Nottingham
- University of Oxford
- Queen Mary University of London
- University of Southampton
- University College London
- University of Warwick
- University of York

**Universities commissioned by Health Education England**

- University of Bath
- London School of Hygiene and Tropical Medicine
- University of Brighton
- University of Swansea
